# Bone and mineral metabolism in patients with primary aldosteronism: A systematic review and meta-analysis

**DOI:** 10.3389/fendo.2022.1027841

**Published:** 2022-10-31

**Authors:** Anning Wang, Yuhan Wang, Hongzhou Liu, Xiaodong Hu, Jiefei Li, Huaijin Xu, Zhimei Nie, Lingjing Zhang, Zhaohui Lyu

**Affiliations:** ^1^ Medical School of Chinese PLA, Beijing, China; ^2^ Department of Endocrinology, The First Medical Center, Chinese PLA General Hospital, Beijing, China; ^3^ Department of Endocrinology, First Hospital of Handan City, Handan, Hebei, China; ^4^ Clinical Medical College, Nankai University, Tianjing, China

**Keywords:** primary aldosteronism, osteoporosis, bone metabolism, secondary hyperparathyroidism (secHPT), meta-analysis

## Abstract

**Purpose:**

Patients with primary aldosteronism (PA) tend to exhibit a high prevalence of osteoporosis (OP) that may vary by whether PA is unilateral or bilateral, and responsive to PA treatment. To explore relationships between bone metabolism, PA subtypes, and treatment outcomes, we performed a systematic review and meta-analysis.

**Methods:**

The PubMed, Embase, and Cochrane databases were searched for clinical studies related to PA and bone metabolism markers. Articles that met the criteria were screened and included in the systematic review; the data were extracted after evaluating their quality. R software (ver. 2022-02-16, Intel Mac OS X 11.6.4) was used for the meta-analysis.

**Results:**

A total of 28 articles were subjected to systematic review, of which 18 were included in the meta-analysis. We found that PA patients evidenced a lower serum calcium level (mean difference [MD] = –0.06 mmol/L, 95% confidence interval [CI]: −0.10 ~ −0.01), a higher urine calcium level (MD = 1.29 mmol/24 h, 95% CI: 0.81 ~ 1.78), and a higher serum parathyroid hormone (PTH) level (MD = 2.16 pmol/L, 95% CI: 1.57 ~ 2.75) than did essential hypertension (EH) subjects. After medical treatment or adrenal surgery, PA patients exhibited a markedly increased serum calcium level (MD = –0.08 mmol/L, 95% CI: –0.11 ~ –0.05), a decreased urine calcium level (MD = 1.72 mmol/24 h, 95% CI: 1.00 ~ 2.44), a decreased serum PTH level (MD = 2.67 pmol/L, 95% CI: 1.73 ~ 3.62), and an increased serum 25-hydroxyvitamin D (25-OHD) level (MD = –6.32 nmol/L, 95% CI: –11.94 ~ –0.70). The meta-analysis showed that the ser um PTH level of unilateral PA patients was significantly higher than that of bilateral PA patients (MD = 0.93 pmol/L, 95% CI: 0.36 ~ 1.49) and the serum 25-OHD lower than that of bilateral PA patients (MD = –4.68 nmol/L, 95% CI: –7.58 ~ 1.77). There were, however, no significant differences between PA and EH patients of 25-OHD, or BMD of femoral neck and lumbar spine. BMDs of the femoral neck or lumbar spine did not change significantly after treatment. The meta-analytical results were confirmed *via* sensitivity and subgroup analyses.

**Conclusion:**

Excess aldosterone was associated with decreased serum calcium, elevated urinary calcium, and elevated PTH levels; these effects may be enhanced by low serum 25-OHD levels. The risks of OP and fracture might be elevated in PA patients, especially unilateral PA patients, but could be reduced after medical treatment or adrenal surgery. In view, however, of the lack of BMD changes, such hypothesis needs to be tested in further studies.

## Introduction

Osteoporosis (OP) is a common disease characterized by decreased bone mass and strength, and destruction of bone microstructure, increasing the fracture susceptibility. OP can be divided into primary and secondary OP. As secondary OP is considered to be reversible, it is important to identify the cause thereof. Secondary OP can develop in those with diseases of the endocrine and blood systems, and of connective tissue; after drug use; and for many other reasons, of which the use of glucocorticoids (1), (2) is the most common. Recently, the role played by mineralocorticoids in bone metabolism has become recognized; an increasing number of studies have shown that primary aldosteronism (PA) may cause secondary OP (3–5). In PA patients, the adrenal cortex autonomously secretes excessive amounts of aldosterone, resulting in sodium retention and potassium excretion, increased blood volume, and suppression of the activity of the renin-angiotensin-aldosterone system (RAAS). This manifests clinically as hypertension and hypokalemia, and is the most common cause of secondary hypertension. Previous studies have shown that PA can affect bone metabolism, and a bidirectional interaction may be in play between aldosterone and parathyroid hormone (PTH) (6, 7). Hence, this study aimed to explore the relationship between bone metabolism markers and the different subtypes of PA, and the effects of PA treatment through a systematic review and meta-analysis.

## Materials and methods

### Search strategy

The PubMed, Embase, and Cochrane databases were searched for clinical studies on PA and bone metabolism markers using the term “Hyperaldosteronism” in combination with “PTH, Calcium, BMD, Osteoporosis, and Secondary Hyperparathyroidism”, with restrictions to the English language and human subjects; the databases were searched from their inceptions to March 6, 2022. We additionally searched the Grey Literature database. The above searches followed the PRISMA guidelines. All retrieved papers were imported into Zotero ver. 6.0.

### Selection criteria

Inclusion criteria: Randomized controlled trials; cohort, case-control, or observational studies. A PA diagnosis verified by at least one confirmatory test (a captopril challenge; a saline infusion, fludrocortisone suppression, or/and an oral sodium loading test); or *via* adrenal venous sampling (AVS). All studies reported the levels of blood parathyroid hormone (PTH), serum calcium and urinary calcium, serum 25-hydroxy vitamin D (25-OHD), serum alkaline phosphatase (AKP), and bone-specific alkaline phosphatase (BAP); the bone mineral density (BMD); and the prevalence or morbidity from OP, fractures, and hyperparathyroidism (HPT).

Exclusion criteria: The PA diagnosis was unclear or not specified. Clinical data were lacking. The experimental or case group, or cohort, were not PA patients. If PA subtypes were studied, there was no clear explanation as to how the subtypes were diagnosed.

### Data extraction and quality assessment

Two researchers independently conducted literature screening and data extraction using a standardized form while operating in a double-blinded manner. We extracted the first author names and countries, type of study, publication year, subject populations, interventions, basic clinical characteristics, bone metabolism markers (biochemical parameters and imaging indices), and follow-up data after PA treatment. The Newcastle-Ottawa Scale (NOS) was used to evaluate the quality of case-control and cohort studies. Any disagreement between the two researchers was settled *via* discussion with, or arbitration by, a third specialist.

### Statistical analysis

R software (ver. 2022-02-16, Intel Mac OS X 11.6.4) (the “meta” package ver. 5.2-0) was used for statistical analysis. We calculated mean differences (MDs) between patients and controls (with 95% confidence limits [CIs]) for continuous variables, and odds ratios (ORs) with 95% CIs for dichotomous variables. We sought heterogeneity using the I^2^ test, and considered that this was absent when I^2^ < 50%. If heterogeneity was absent, we used fixed-effect models for meta-analysis, but employed random-effect models when heterogeneity was in play and the causes thereof were not discernible. Publication bias was assessed using the Egger’s test; we also drew funnel plots during meta-analyses of at least 10 studies. Visual confirmation of funnel plot symmetry, or an Egger’s test P-value > 0.05, indicated no publication bias. We performed sensitivity analysis on only “high quality” studies (NOS scores at least the median of all studies). We also performed subgroup analyses to explore the sources of heterogeneity.

## Results

### Search results

**Figure 1:**
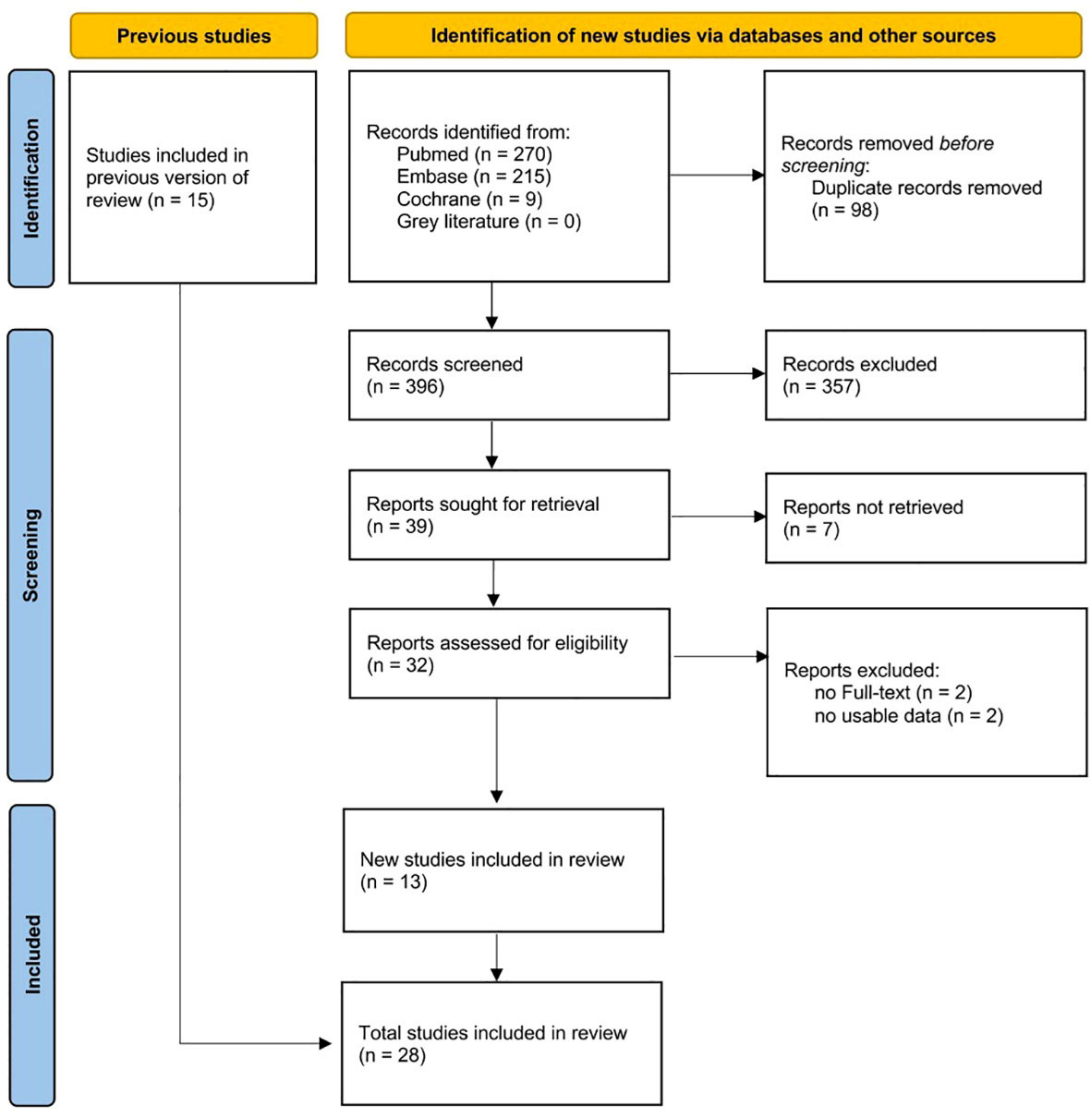
Flow diagram of literature selection.

A total of 494 articles were retrieved, including 270 from PubMed, 215 from Embase, 9 from Cochrane, but none from the Grey Literature (**Figure 1**). After removing duplicate studies, reading the titles, and screening the full texts, 28 works were finally included in the systematic review (**Table 1**), of which 13 were newly included compared to a previous study (3). Eighteen articles were included in the meta-analysis. In detail, 12 studies (14, 17, 18, 21, 25–27, 29–33) compared 3,318 PA patients to 11,024 essential hypertension (EH) subjects in terms of the serum calcium and urinary calcium levels; the serum PTH, 25-OHD, and AKP levels; and the BMD. Twelve studies (17), (18), (25), (27, 29–31), (12, 15, 16, 28, 33) compared 501 PA patients before medical or surgical treatment to 491 who were followed-up after treatment in terms of the serum calcium and urinary calcium levels; the serum PTH, 25-OHD, and BAP levels; and the BMD. Eight studies (nine datasets) (14), (25), (26), (29), (15), (11, 13, 19) compared 489 unilateral PA patients with 558 bilateral PA patients (three studies diagnosed PA *via* AVS after ACTH stimulation; four *via* AVS without ACTH stimulation; and one *via* AVS both with and without ACTH stimulation); all presented the serum calcium and urinary calcium and PTH and 25-OHD levels; and the BMD.

**Table 1:** Characteristic of studies included in systematic review and meta-analysis.

Study	Design	Country	Patient and Control	N	age (year)	Male (%)	NOS scores
Zavatta, 2022 (8)	case-control	Italy	PA NFA	26 39	58.00 ± 8.90 59.00 ± 8.80	11 (42.3%) 17 (43.6%)	8
Tang, 2022 (9)	case-control	China	PA SA(GS+BS)	20 37	38.80 ± 10.10 36.10 ± 14.60	11 (55.0%) 19 (51.4%)	7
Liu, 2021 (10)	case-control	China	PA NFA	356 417	50.00 ± 11.00 51.00 ± 12.00	203 (57.0%) 219 (52.5%)	6
Kometani, 2021 (11)	case-control	Japan	(a) unilateral PA bilateral PA (without ACTH)	60 54	52.00 ± 12.00 52.00 ± 12.00	31 (51.7%) 27 (50.0%)	6
			(b) unilateral PA bilateral PA (with ACTH)	19 105	55.00 ± 11.00 51.00 ± 11.00	12 (63.2%) 52 (49.5%)	
Gravvanis, 2021 (12)	case-control	Greece	PA before treatment PA after treatment	63 63	60.00(31.00,78.00) 60.00(31.00,78.00)	37 (58.7%) 37 (58.7%)	8
Yokomoto, 2020 (13)	case-control	Japan	unilateral PA bilateral PA (with ACTH)	37 76	56.00 ± 14.00 54.00 ± 11.00	25 (67.6%) 23 (30.3%)	7
Tuersun, 2020 (14)	case-control	China	(a) PA EH	156 156	49.86 ± 8.72 48.99 ± 8.60	88 (56.4%) 88 (56.4%)	8
			(b) unilateral PA bilateral PA (without ACTH)	76 80	49.11 ± 8.38 48.79 ± 8.86	43 (56.6%) 47 (58.8%)	
Asbach, 2020 (15)	case-control	German	(a) unilateral PA bilateral PA (with ACTH)	70 55	49.00(43.00,59.00) 45.00(41.00,53.00)	44 (62.9%) 35 (63.6%)	8
			(b) PA before treatment PA after treatment	60 60	- -	38 (63.3%) 38 (63.3%)	
Adolf, 2020 (16)	case-control	German	(a) PA HS	36 18	59.00(53.00,64.00) 54.00(44.00,60.00)	0 (0%) 0 (0%)	8
			(b) PA before treatment PA after treatment	36 36	59.00(53.00,64.00) 59.00(53.00,64.00)	0 (0%) 0 (0%)	
Lenzini, 2019 (17)	case-control	Italy	(a) PA EH	42 63	52.00 ± 11.22 52.00 ± 10.00	24 (57.1%) 33 (52.4%)	7
			(b) unilateral PA bilateral PA	27 15	52.00 ± 11.00 52.00 ± 12.00	15 (55.6%) 9 (60.0%)	
			(c) PA before treatment PA after treatment	42 32	52.00 ± 11.22 52.00 ± 11.02	24 (57.1%) -	
Loh, 2018 (18)	case-control	Malaysia	(a) PA EH	18 17	50.00(38.00,58.75) 50.00(38.50,61.50)	11 (61.1%) 11 (64.7%)	7
			(b) PA before treatment PA after treatment	15 15	- -	- -	
Lim, 2018 (19)	case-control	Korean	unilateral PA bilateral PA (with ACTH)	23 19	46.50 ± 11.50 51.50 ± 9.80	9 (39.1%) 12 (63.2%)	8
Kim, 2018 (5)	case-control	Korean	PA AI	72 335	56.73 ± 8.50 54.64 ± 9.95	- -	8
Shu, 2018 (20)	case-control	China	OP OE HS	186 96 42	59.60 ± 4.35 58.70 ± 4.40 58.60 ± 4.90	0 (0%) 0 (0%) 0 (0%)	8
Wu, 2017 (21)	cohort	China	PA EH	2533 10132	50.55 ± 14.57 50.69 ± 17.90	1176 (46.4%) 4708 (46.5%)	8
Salcuni, 2017 (22)	case-control	Italy	(a) PA non-PA	12 310	60.40 ± 13.50 61.10 ± 9.30	- -	9
			(b) OP HS	213 109	- -	- -	
Notsu, 2017 (23)	case-control	Japan	PA HS	56 56	58.70 ± 11.10 59.40 ± 11.50	25 (44.6%) 25 (44.6%)	8
Zhang, 2016 (24)	case-control	China	PA NFA	84 58	50.00 ± 10.00 55.00 ± 8.00	44 (52.4%) 21 (36.2%)	6
Jiang, 2016 (25)	case-control	China	(a) PA EH	242 120	49.00(41.00,57.00) 50.00(42.00,58.00)	131 (54.1%) 53 (44.2%)	8
			(b) unilateral PA bilateral PA (without ACTH)	123 119	46.57 ± 11.33 52.70 ± 12.01	57 (46.3%) 74 (62.2%)	
			(c) PA before treatment PA after treatment	99 99	- -	- -	
Petramala, 2014 (26)	case-control	Italy	(a) PA EH HS	73 73 40	52.50 ± 11.20 55.60 ± 12.40 55.70 ± 6.10	- - -	6
			(b) unilateral PA bilateral PA (without ACTH)	35 38	52.80 ± 11.50 52.50 ± 11.20	- -	
Ceccoli, 2013 (27)	case-control	Italy	(a) PA EH	116 110	51.60 ± 11.00 55.00 ± 10.00	65 (56.0%) 35 (31.8%)	7
			(b) PA before treatment PA after treatment	40 40	- -	- -	
Salcuni, 2012 (28)	case-control	Italy	(a) PA NFA	11 15	56.00 ± 9.30 56.70 ± 9.50	4 (36.4%) 5 (33.3%)	10
			(b) PA before treatment PA after treatment	9 9	- -	- -	
Rossi, 2012 (29)	case-control	Italy	(a) PA EH	58 74	49.76 ± 12.60 50.00 ± 14.00	- -	9
			(b) unilateral PA bilateral PA (without ACTH)	46 12	51.00 ± 13.00 45.00 ± 10.00	- -	
			(c) PA before treatment PA after treatment	46 46	51.00 ± 13.00 52.00 ± 12.00	- -	
Pilz, 2012 (30)	case-control	German	(a) PA EH	10 182	50.10 ± 11.00 50.20 ± 15.70	4 (40.0%) 74 (40.7%)	9
			(b) PA before treatment PA after treatment	10 10	50.10 ± 11.00 51.20 ± 11.50	4 (40.0%) 4 (40.0%)	
Maniero, 2012 (31)	case-control	Italy	(a) PA EH	44 61	50.00 ± 13.00 50.00 ± 15.00	18 (40.9%) 21 (34.4%)	9
			(b) PA before treatment PA after treatment	31 31	- -	- -	
Rossi, 1998 (32)	case-control	Italy	PA EH	16 16	50.80 ± 2.70 48.50 ± 2.30	8 (50.0%) 8 (50.0%)	7
Rossi, 1995 (33)	case-control	Italy	(a) PA EH HS	10 20 10	52.40 ± 12.90 46.00 ± 7.19 48.00 ± 12.70	5 (50.0%) 10 (50.0%) 5 (50.0%)	6
			(b) PA before treatment PA after treatment	14 14	- -	- -	
Lawrence, 1985 (34)	descriptive	American	PA	10	–	–	–

N, number; NOS, Newcastle-Ottawa Scale; PA, primary aldosteronism; NFA, non-functioning adrenal tumour; SA, secondary aldosteronism; GS, gitelman syndrome; BS, bartter syndrome; AI, adrenal incidentaloma; OP, osteoporosis; OE, osteopenia; HS, healthy subjects.

### Quality assessment

Of the 28 included articles, 26 were case-control studies, one a cohort study, and one a descriptive study. The quality of the case-control studies is shown in **Supplementary Table 3**; the NOS score of the cohort study was 8. The NOS scores of all studies were at least 6 (median 8).

### PA vs. EH

**Figure 2:**
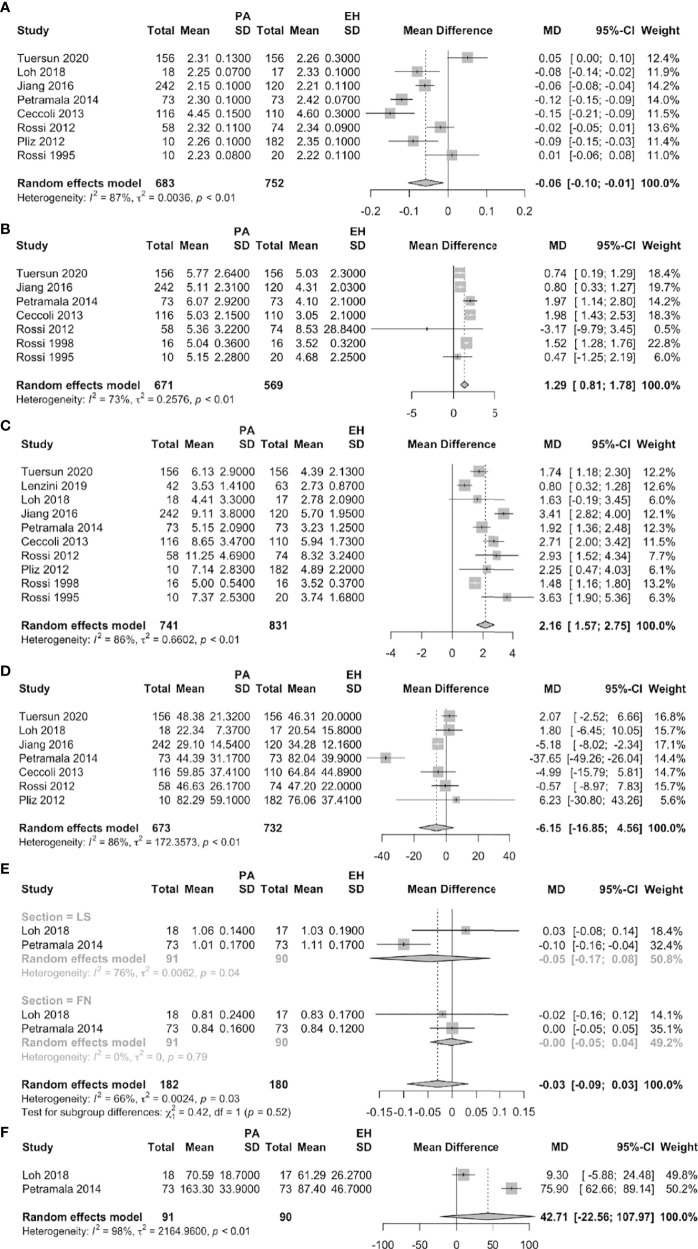
Forest plots of PA vs. EH patients (**A**: serum calcium, **B**: urine calcium, **C**: serum PTH, **D**: serum 25-OHD, **E**: BMD of FN and LS, **F**: AKP).

Eight studies reported significantly lower serum calcium levels in PA than EH patients (MD = –0.06 mmol/L, 95% CI: –0.10 ~ –0.01). The urinary calcium level was significantly higher in seven studies that compared PA patients to EH subjects (MD = 1.29 mmol/24 h, 95% CI: 0.81 ~ 1.78). Ten studies found that the serum PTH level was significantly higher in PA patients than in EH subjects (MD = 2.16 pmol/L, 95% CI: 1.57 ~ 2.75). While no significant differences were found between PA and EH patients on 25-OHD, on BMD of femoral neck and lumbar spine and on AKP (**Figure 2**).

### A comparison of data before and after treatment

**Figure 3:**
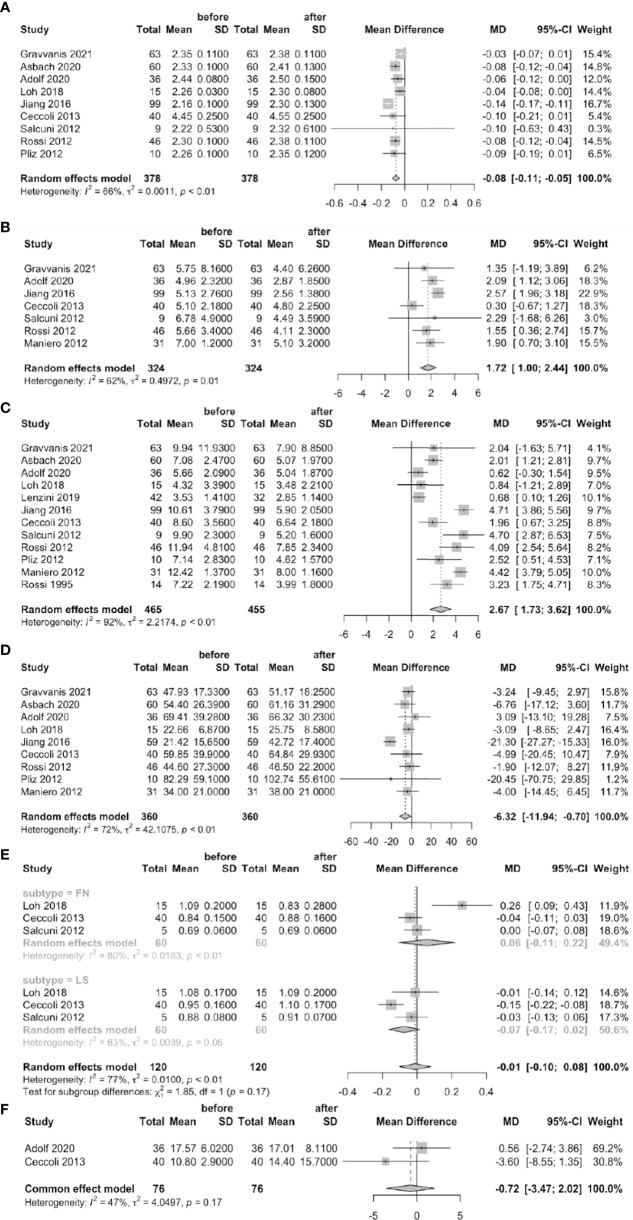
Forest plots of before-treatment vs. after-treatment PA patients (**A**: serum calcium, **B**: urine calcium, **C**: serum PTH, **D**: serum 25-OHD, **E**: BMD of FN and LS, **F**: BAP).

Nine studies reported significantly higher serum calcium levels (MD = –0.08 mmol/L, 95% CI: –0.11 ~ –0.05) after than before treatment of PA patients. The urinary calcium level was significantly lower after treatment (MD = 1.72 mmol/24 h, 95% CI: 1.00 ~ 2.44) in seven studies; the serum PTH level was significantly lower in 12 studies (MD = 2.67 pmol/L, 95% CI: 1.73 ~ 3.62); and the serum 25-OHD was significantly higher (MD = –6.32 nmol/L, 95% CI: –11.94 ~ –0.70) in nine studies. None of the BAP level or the BMDs of the femoral neck or lumbar spine changed significantly after treatment (**Figure 3**).

### Unilateral vs. bilateral PA

**Figure 4:**
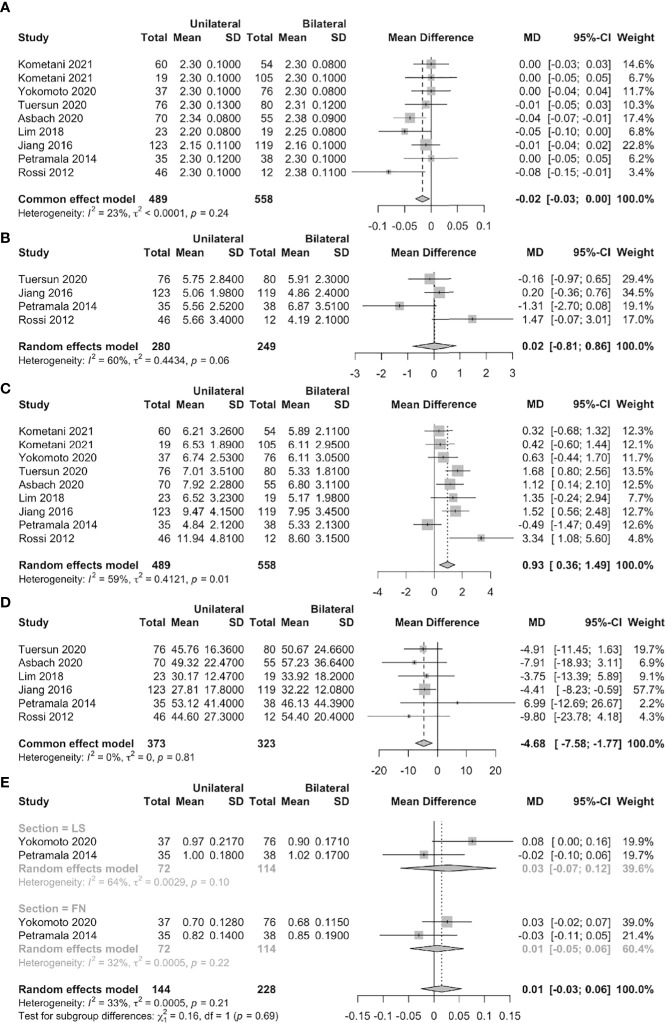
Forest plots of unilateral vs. bilateral PA patients (**A**: serum calcium, **B**: urine calcium, **C**: serum PTH, **D**: serum 25-OHD, **E**: BMD of FN and LS).

The serum PTH level was significantly higher in unilateral than bilateral PA patients (MD = 0.93 pmol/L, 95% CI: 0.36 ~ 1.49) in eight studies (nine datasets). Six studies found that the serum 25-OHD level was significantly lower in unilateral than bilateral PA patients (MD = –4.68 nmol/L, 95% CI: –7.58 ~ –1.77). However, no significant differences were found in terms of the serum calcium or urinary calcium level or the BMDs of the femoral neck and lumbar spine (**Figure 4**).

### Publication bias

**Figure 5:**
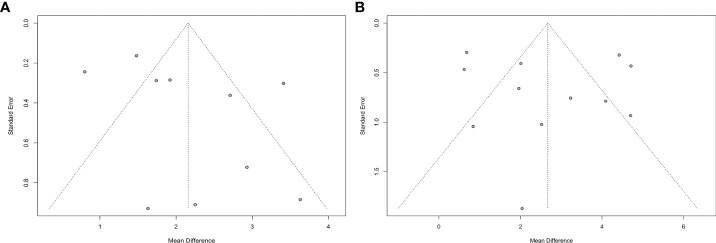
Funnel plots of meta-analysis on PTH (**A**: PA vs. EH, **B**: Before-treatment vs. After-treatment PA patients).

Publication bias in terms of PTH levels was sought in works that compared PA patients to EH subjects and PA patients before and after treatment. The funnel plots were substantially symmetrical, and the Egger’s test revealed no obvious publication bias (**Figure 5**).

### Sensitivity analysis

The median NOS score was 8. Thus, we repeated the analysis including only “high quality” studies (NOS ≥ 8). None of the AKP or BAP level, or the BMD, were included in sensitivity analysis because too few studies reported such data. After excluding “low quality” studies, the above data on urinary calcium and serum PTH levels were largely confirmed. In contrast, no significant differences were found between PA patients and EH subjects in terms of serum calcium levels, or between PA patients before and after treatment in terms of serum 25-OHD levels. However, the serum calcium level differed significantly between unilateral and bilateral PA patients (**Table 2**).

**Table 2:** Sensitivity analysis on “high quality” studies (NOS≥8).

Item	N of studies	N of patients/controls	MD (95% CI)	I^2^	P-value
***PA vs. EH***					
Serum calcium	4	466/532	-0.03 (-0.09, 0.03)*	84%	<0.01
Urine calcium	3	456/350	0.76 (0.41, 1.12)	0%	0.50
Serum PTH	4	466/532	2.59(1.69, 3.50)	82%	<0.01
Serum 25-OHD	4	466/532	-1.51 (-6.43, 3.41)	60%	0.06
***Before-treatment vs. After-treatment PA***					
Serum calcium	7	323/323	-0.08 (-0.11, -0.05)	70%	<0.01
Urine calcium	6	284/284	2.17 (1.70, 2.65)	0%	0.66
Serum PTH	8	354/354	3.19 (2.03, 4.36)	90%	<0.01
Serum 25-OHD	7	305/305	-7.00 (-14.13, 0.13)*	76%	<0.01
***Unilateral vs. Bilateral PA***					
Serum calcium	5	338/285	-0.03 (-0.04, -0.01)*	35%	0.19
Urine calcium	3	245/211	0.20 (-0.24, 0.64)	40%	0.19
Serum PTH	5	338/285	1.54 (1.04, 2.04)	0%	0.51
Serum 25-OHD	5	338/285	-4.94 (-7.87, -2.00)	0%	0.93

N, number; MD, mean difference; 95% CI, 95% Conﬁdence Intervals; * MD results changed after excluding “low quality” studies.

### Subgroup analysis

All of study location (Asia or Europe), the assays used to measure serum PTH and 25-OHD, and the AVS procedure (with or without ACTH stimulation) might have affected our assessments; we thus performed three subgroup analyses. At least one such analysis eliminated the heterogeneity, except in terms of the serum calcium, PTH, and 25-OHD comparisons between PA patients and EH subjects (**Table 3**).

**Table 3:** Subgroup analyses of study location, the assay used to measure serum PTH and 25-OHD, and the AVS procedure.

Item	Subgroup	N of studies	N of patients	MD (95% CI)	I^2^	P-value for I^2^	X^2^
***PA vs. EH***							
Serum calcium	Asian Europe	3 5	416/293 267/459	-0.03 (-0.11, 0.05) -0.07 (-0.13, -0.02)	88% 87%	<0.01 <0.01	0.81
Urine calcium	Asian* Europe	2 5	398/276 273/293	0.77 (0.42, 1.13) 1.59 (1.39, 1.80)	0% 40%	0.87 0.15	15.19
Serum PTH	1. Asian Europe	3 7	416/293 325/538	2.37 (1.14, 3.59) 2.05 (1.36, 2.75)	88% 81%	<0.01 <0.01	0.19
	2. ECLIA ELISA CLIA RIA Mix	3 1 2 3 1	282/448 42/63 260/137 99/109 58/74	2.20 (1.46, 2.94) 0.80 (0.32, 1.28) 2.74 (1.04, 4.43) 2.02 (1.12, 2.92) 2.93 (1.52, 4.34)	55% - 70% 71% -	0.11 - 0.07 0.03 -	18.9
Serum 25-OHD	1. Asian Europe	3 4	416/293 257/439	-0.16 (-6.34, 4.21) -11.15 (-30.75, 8.45)	76% 89%	0.02 <0.01	0.95
	2. ECLIA ECLA CLA	1 2 4	156/156 260/137 257/439	2.07 (-2.52, 6.66) -2.81 (-9.29, 3.67) -11.15 (-30.75, 8.45)	- 59% 89%	- 0.12 <0.01	2.75
***Before-treatment vs. After-treatment PA***							
Serum calcium	Asian Europe*	2 7	378/378 264/264	-0.09 (-0.19, 0.01) -0.06 (-0.09, -0.04)	92% 0%	<0.01 0.56	0.26
Urine calcium	Asian Europe*	1 6	99/99 225/225	2.57 (1.96, 3.18) 1.46 (0.71, 2.20)	- 36%	- 0.17	5.16
Serum PTH	1. Asian Europe	2 10	114/114 351/341	2.89 (-0.89, 6.68) 2.58 (1.60, 3.57)	91% 91%	<0.01 <0.01	0.01
	2. ECLIA* CLA CLIA ELISA Mix RIA	4 1 4 1 1 1	173/173 36/36 154/154 42/32 46/46 14/14	2.05 (1.42, 2.68) 0.62 (-0.30, 1.54) 3.83 (2.22, 5.44) 0.68 (0.10, 1.26) 4.09 (2.54, 5.64) 3.23 (1.75, 4.71)	0% - 75% - - -	0.97 - <0.01 - - -	37.87
Serum 25-OHD	1. Asian Europe*	2 7	74/74 286/286	-12.16 (-30.01, 5.68) -3.51 (-7.51, 0.49)	95% 0%	<0.01 0.95	0.86
	2. ELISA CLA* ECLIA ECLA RIA	1 5 1 1 1	63/63 192/192 15/15 59/59 31/31	-3.24 (-9.45, 2.97) -3.60 (-9.64, 2.44) -3.09 (-8.65, 2.47) -21.30 (-27.27, -15.33) -4.00 (-14.45, 6.45)	- 0% - - -	- 0.81 - - -	26.77
***Unilateral vs. Bilateral PA***							
Serum calcium **#**	1. Asian* Europe	6 3	338/453 151/105	-0.01 (-0.01, 0.01) -0.04 (-0.06, -0.01)	0% 44%	0.64 0.17	3.38
	2. With ACTH Without ACTH	4 5	149/255 340/303	-0.02 (-0.04, 0.00) -0.01 (-0.03, 0.01)	36% 11%	0.19 0.34	1.11
Urine calcium	Asian* Europe	2 2	199/199 81/81	0.09 (-0.37, 0.54) 0.06 (-2.66, 2.78)	0% 85%	0.47 <0.01	0
Serum PTH	1. Asian* Europe	6 3	338/453 151/105	0.99 (0.46, 1.51) 0.81 (-0.50, 2.12)	29% 83%	0.22 <0.01	0.02
	2. With ACTH* Without ACTH	4 5	149/255 340/303	1.12 (-0.90, 3.14) 1.09 (-0.01, 2.19)	0% 78%	0.68 <0.01	0.19
	3. ECLIA* RIA CLIA Mix	5 2 1 1	262/370 58/57 123/119 46/12	0.87 (0.34, 1.40) 0.32 (-1.47, 2.11) 1.52 (0.56, 2.48) 3.34 (1.08, 5.60)	28% 73% - -	0.23 0.05 - -	5.94
Serum 25-OHD **#**	1. Asian* Europe*	3 3	222/218 151/105	-4.45 (-7.58, -1.33) -6.10 (-14.03, 1.82)	0% 3%	0.98 0.35	0.14
	2. With ACTH* Without ACTH*	2 4	93/74 280/249	-5.55 (-12.81,1.70) -4.51 (-7.68,-1.34)	0% 0%	0.58 0.60	0.07
	3. ECLIA CLA* ECLA Unknown	1 3 1 1	76/80 151/105 123/119 23/19	-4.91 (-11.45, 1.63) -6.10 (-14.03, 1.82) -3.75 (-13.39, 5.89) 04.41 (-8.23, -0.59)	- 3% - -	- 0.35 - -	0.18

N, number; MD, mean difference; 95% CI, 95% Conﬁdence Intervals; CLA, chemiluminescence assay; ECLA, electro-chemiluminescence assay; ECLIA, electro-chemiluminescence immunoassay; ELISA, enzyme linked immunosorbent assay; CLIA, chemiluminescent immunoassay; RIA, immunoradiometric assays; *I^2^<50% in subgroup analysis; **#** I^2^<50% before subgroup analysis.

## Discussion

We consistently found that PA patients (especially unilateral patients) were at higher risk of OP than EH subjects but the risk was reduced after medical treatment or surgery. Thus, aldosterone may affect both PTH secretion and bone metabolism.

### Direct effects of aldosterone on bone metabolism

Excess aldosterone directly affects bone formation and resorption; inhibition of the RAAS system reduces bone loss (17). The following mechanisms may explain these phenomena. The long-term oxidative stress and chronic inflammation of PA patients may increase osteoblast and osteocyte apoptosis (35), triggering abnormal bone metabolism, secondary OP, and even fracture (36), (37). It is well known that glucocorticoids can affect bone metabolism. Recently, mineralocorticoids have also been found to be related to bone metabolism. Researchers have found that MR antagonists (MRAs) reduced the risk of fracture in patients with secondary aldosteronism (38). Beavan et al. (39) reported (in 2001) that MR existed in human osteocytes; aldosterone may thus act directly on osteocytes. Later studies showed that several genes of the mineralocorticoid pathway (NR3C2, PIK3R1, PRKCH, and SCNN1B) may affect bone strength (40).

### Aldosterone may affect bone metabolism by interacting with PTH or vitamin D

Resnick et al. (34) were the first to report elevated PTH levels in PA patients (in 1985). Many studies (14, 17, 18), (25–27, 29, 30), (32), (33) have confirmed this. A possible bidirectional interaction between aldosterone and PTH has been suggested (6), (7). Several studies have proposed that aldosteronism may cause secondary HPT by reducing sodium and calcium re-absorption by the proximal renal tubules (26), (27), (30), (32), increasing urinary calcium excretion, and long-term calcification. This explains why PA patients tend to have higher serum calcium but lower urinary calcium levels than others. It has been suggested that, in the acute phase, regulation of PTH secretion by the RAAS system is mediated by angiotensin II (Ang II), but aldosterone may be involved in such regulation in the chronic phase (41). PTH and calcium levels both affect aldosterone secretion; however, that is not our topic here.

PA patients more commonly exhibit vitamin D deficiency than others (42); vitamin D supplementation downregulates the RASS system (43), (44). We found no significant difference in serum 25-OHD levels between PA and EH patients, but the level increased significantly in PA patients after medical treatment or surgery, suggesting that vitamin D might be involved in the action of aldosterone in terms of bone metabolism. Vitamin D increases the intestinal absorption of calcium and phosphorus, re-absorption of calcium and phosphate in the renal tubules, and bone calcium deposition. Reduced levels of serum vitamin D stimulate PTH secretion and upregulate the RAAS system (44–48); the 1,25(OH)_2_D receptor complex inhibits the expression of renin *in vitro* (43), (49), (50). In PA patients, the level of the vitamin D receptor (VDR) may be associated with that of a marker of osteoclast activation, thus tartrate-resistant acid phosphatase 5b (TRACP-5b) (6). Fibroblast growth factor 23 (FGF23) is a phosphorylated protein regulated by phosphate and 1,25(OH)_2_D. The latter stimulates the production of FGF23 and creates a negative feedback loop that regulates the production itself (51), as well as PTH secretion. FGF23 plays roles in PA-related HPT and improved parathyroid function after adrenalectomy in PA patients (52), but vitamin D supplementation does not completely correct HPT in PA patients (31), (10). Low vitamin D levels may (together with high aldosterone levels) affect PTH secretion and bone metabolism in PA patients, increasing the OP risk.

### Risk of OP in PA patients

After excluding the effect of hypertension (23), PA patients still had a higher fracture risk than general populations (28), (13), (23), (22). Wu et al. (53) confirmed a high prevalence of such fractures in a prospective cohort study. The BMD is commonly used to evaluate bone strength. We found no significant difference in the BMD of either the lumbar spine or femoral neck before and after PA treatment. We did not meta-analyze the BMD data of works that compared PA and EH groups because there were few such studies. However, Petramala et al. (26) and Salcuni et al. (28) found that the osteopenia and OP rates in PA patients were significantly higher than in EH patients, healthy subjects, and those with adrenal non-functioning tumors (NFAs). This was confirmed by another study comparing patients with PA and secondary aldosteronism (9).

The trabecular bone score (TBS) is a new indicator of bone microstructure, used to evaluate bone quality and fracture risk. Several studies have suggested that aldosterone may trigger osteopenia by destroying bone microstructure rather than by reducing the BMD (5), (54); the TBS may be a better indicator than the BMD when screening for OP in PA patients. Although no significant BMD changes were evident in early-stage PA patients, bone turnover increased (as revealed by changes in the CTX and PINP levels) (18). Unfortunately, no study has used the TBS to compare EH and PA patients.

The effect of aldosterone on BMD remains controversial (28), (23), and the mechanism of that has not been fully elucidated. Furthermore, more research is needed on how aldosterone may affect bone metabolism and the possible association between TBS and aldosterone in the future.

### Does bone metabolism differ between unilateral and bilateral PA patients?

This possibility remains controversial (14), (17), (25), (26), (29), (15), (11, 13, 19), (55), (56). All except three studies that we reviewed found that the serum PTH levels differed between unilateral and bilateral PA patients; the exceptions were Kometani et al., Jiang et al., and Riester et al. (25), (11), (55). Petramala et al. (26) found that patients with aldosterone-producing adenomas (APAs) exhibited more bone remodeling than did those with idiopathic hyperaldosteronism (IHA). Yokomoto et al. (13) reported that unilateral PA was an independent risk factor for vertebral fracture. We found that, in patients with unilateral PA, the serum PTH level was higher and the serum 25-OHD level lower than in patients with bilateral PA, which meant that a high serum aldosterone level is associated with a high serum PTH level, indicating that unilateral PA patients are at higher risk of sHPT.

### Strengths and limitations

There were 2 meta-analyses before our research. Loh et al. (4) published a conference abstract in 2019, meta-analyzing the difference between PA (n=352) and non-PA (n=587) patients, and between before and after treatment in PA patients. Shi et al. (3) just meta-analyzed the difference between PA and EH patients with 15 articles in 2020. Unlike previous work (3), (4), we are the first to review systematically and meta-analyze the possible effects of PA subtypes on bone metabolism. The number of included papers and the sample size of the present study are larger than those of the previous work. However, certain limitations of our work should be acknowledged. First, most included studies were observational and had small patient numbers. Second, the assays used to measure the levels of plasma aldosterone, and serum PTH and 25-OHD, varied. Third, some studies evidenced heterogeneity. Although the sensitivity analyses eliminated most heterogeneity, a possibility of bias remains. Fourth, few works dealt with the effects of different PA subtypes on BMD; we could engage in only descriptive analyses.

## Conclusion

Excess aldosterone was associated with decreased serum calcium, elevated urinary calcium, and elevated PTH levels; these effects may be enhanced by low serum 25-OHD levels. The risks of OP and fracture might be elevated in PA patients, especially unilateral PA patients, but could be reduced after medical treatment or adrenal surgery. The lack of BMD changes after treatment, indicates, however, that PTH and calcium changes may also represent an epiphenomenon of limited clinical significance, so more research is needed, either to confirm, or to refute the notion of any significant change in fracture rate in PA patients. TBS may be a better indicator than BMD when screening for OP in PA patients. More research is needed on how aldosterone may affect bone metabolism and the possible association between TBS and aldosterone.

## Data availability statement

The original contributions presented in the study are included in the article/**Supplementary Material**. Further inquiries can be directed to the corresponding author.

## Author contributions

AW and ZL designed the study. HL and XH collected the data. AW performed the meta-analysis and drafted the manuscript. JL, HX, ZN and LZ partially planned the research. ZL edited the manuscript. All authors contributed to the article and have approved the submitted version.

## Conflict of interest

The authors declare that the research was conducted in the absence of any commercial or financial relationships that could be construed as a potential conflict of interest.

## Publisher’s note

All claims expressed in this article are solely those of the authors and do not necessarily represent those of their affiliated organizations, or those of the publisher, the editors and the reviewers. Any product that may be evaluated in this article, or claim that may be made by its manufacturer, is not guaranteed or endorsed by the publisher.

## Supplementary material

Supplementary Figure 1 Subgroup analysis of PA and EH (**A**: serum calcium, **B**: urine calcium, **C**: serum PTH, **D**: serum 25-OHD).

Supplementary Figure 2 Subgroup analysis of before-treatment and after-treatment PA patients (**A**: serum calcium, **B**: urine calcium, **C**: serum PTH, **D**: serum 25-OHD).

Supplementary Figure 3 Subgroup analysis of unilateral and bilateral PA patients (**A**: serum calcium, **B**: urine calcium, **C**: serum PTH, **D**: serum 25-OHD).

## References

[1] Canalis E Mazziotti G Giustina A Bilezikian JP . Glucocorticoid-induced osteoporosis: pathophysiology and therapy. Osteoporos Int (2007) 18(10):1319–28. doi: 10.1007/s00198-007-0394-0 17566815

[2] Rizzoli R Biver E . Glucocorticoid-induced osteoporosis: who to treat with what agent? Nat Rev Rheumatol (2015) 11(2):98–109. doi: 10.1038/nrrheum.2014.188 25385412

[3] Shi S Lu C Tian H Ren Y Chen T . Primary aldosteronism and bone metabolism: A systematic review and meta-analysis. Front Endocrinol (Lausanne) (2020) 11:574151. doi: 10.3389/fendo.2020.574151 33101208 PMC7546890

[4] Loh HH Yee A Loh HS . Bone health among patients with primary aldosteronism: a systematic review and meta-analysis. Minerva Endocrinol (2019) 44(4):387–96. doi: 10.23736/S0391-1977.18.02867-5 30482008

[5] Kim B-J Lee SH Koh J-M . Bone health in adrenal disorders. Endocrinol Metab (2018) 33(1):1–8. doi: 10.3803/EnM.2018.33.1.1 PMC5874185 29589383

[6] Gao X Yamazaki Y Tezuka Y Onodera Y Ogata H Omata K . The crosstalk between aldosterone and calcium metabolism in primary aldosteronism: A possible calcium metabolism-associated aberrant ‘neoplastic’ steroidogenesis in adrenals. J Steroid Biochem Mol Biol (2019) 193:105434. doi: 10.1016/j.jsbmb.2019.105434 31351131

[7] Brown JM Vaidya A . Interactions between adrenal-regulatory and calcium-regulatory hormones in human health. Curr Opin Endocrinol Diabetes Obes (2014) 21(3):193–201. doi: 10.1097/MED.0000000000000062 24694551 PMC4123208

[8] Zavatta G Di Dalmazi G Altieri P Pelusi C Golfieri R Mosconi C . Association between aldosterone and parathyroid hormone levels in patients with adrenocortical tumors. Endocr Pract (2022) 28(1):90–5. doi: 10.1016/j.eprac.2021.09.002 34508903

[9] Tang W Chai Y Jia H Wang B Liu T Wang H . Different roles of the RAAS affect bone metabolism in patients with primary aldosteronism, gitelman syndrome and bartter syndrome. BMC Endocr Disord (2022) 22(1) : 38. doi: 10.1186/s12902-022-00955-2 PMC8840772 35148746

[10] Liu Y Yang G Pei Y Dou J Lyu Z Du J . Value of serum parathyroid hormone in the diagnosis of primary aldosteronism. Nat Med J China (2021) 101(34):2674–80. doi: 10.3760/cma.j.cn112137-20210111-00088 34510873

[11] Kometani M Yoneda T Aono D Gondoh-Noda Y Matsuoka T Higashitani T . Primary aldosteronism with parathyroid hormone elevation: A single-center retrospective study. Intern Med (2021) 60(7):993–8. doi: 10.2169/internalmedicine.5282-20 PMC8079911 33790140

[12] Gravvanis C Papanastasiou L Glycofridi S Voulgaris N Tyfoxylou E Theodora K . Hyperparathyroidism in patients with overt and mild primary aldosteronism. Hormones (Athens) (2021) 20(4):793–802. doi: 10.1007/s42000-021-00319-w 34524646

[13] Yokomoto-Umakoshi M Sakamoto R Umakoshi H Matsuda Y Nagata H Fukumoto T . Unilateral primary aldosteronism as an independent risk factor for vertebral fracture. Clin Endocrinol (2020) 92(3):206–13. doi: 10.1111/cen.14145 31868939

[14] Tuersun T Luo Q Zhang Z Wang G Zhang D Wang M . Abdominal aortic calcification is more severe in unilateral primary aldosteronism patients and is associated with elevated aldosterone and parathyroid hormone levels. Hypertens Res (2020) 43(12):1413–20. doi: 10.1038/s41440-020-0529-7 32770102

[15] Asbach E Bekeran M König A Lang K Hanslik G Treitl M . Primary and secondary hyperparathyroidism in patients with primary aldosteronism - findings from the German conn’s registry. Exp Clin Endocrinol Diabetes (2020) 128(4):246–54. doi: 10.1055/a-1027-6472 31698477

[16] Adolf C Braun LT Fuss CT Hahner S Künzel H Handgriff L . Spironolactone reduces biochemical markers of bone turnover in postmenopausal women with primary aldosteronism. Endocrine (2020) 69(3):625–33. doi: 10.1007/s12020-020-02348-8 PMC8514385 32594379

[17] Lenzini L Prisco S Vanderriele PE Lerco S Torresan F Maiolino G . PTH modulation by aldosterone and angiotensin II is blunted in hyperaldosteronism and rescued by adrenalectomy. J Clin Endocrinol Metab (2019) 104(9):3726–34. doi: 10.1210/jc.2019-00143 30865228

[18] Loh HH Kamaruddin NA Zakaria R Sukor N . Improvement of bone turnover markers and bone mineral density following treatment of primary aldosteronism. Minerva Endocrinol (2018) 43(2):117–25. doi: 10.23736/S0391-1977.16.02553-0 28001017

[19] Lim JS Hong N Park Park S Park S II Oh YT Yu MH . Effects of altered calcium metabolism on cardiac parameters in primary aldosteronism. Endocrinol Metab (2018) 33(4):485–92. doi: 10.3803/EnM.2018.33.4.485 PMC6279903 30513563

[20] Shu X Mei M Ma L Wang Z Yang S Hu J . Postmenopausal osteoporosis is associated with elevated aldosterone/renin ratio. J Hum Hypertens (2018) 32(7):524–30. doi: 10.1038/s41371-018-0069-7 29789689

[21] Wu V-C Chang C-H Wang C-Y Lin Y-H Kao T-W Lin P-C . Risk of fracture in primary aldosteronism: A population-based cohort study. J Bone Miner Res (2017) 32(4):743–52. doi: 10.1002/jbmr.3033 27862274

[22] Salcuni AS Carnevale V Battista C Palmieri S Eller-Vainicher C Guarnieri V . Primary aldosteronism as a cause of secondary osteoporosis. Eur J Endocrinol (2017) 177(5):431–7. doi: 10.1530/EJE-17-0417 28794160

[23] Notsu M Yamauchi M Yamamoto M Nawata K Sugimoto T . Primary aldosteronism as a risk factor for vertebral fracture. J Clin Endocrinol Metab (2017) 102(4):1237–43. doi: 10.1210/jc.2016-3206 28182819

[24] Zhang L-X Gu W-J Li Y-J Wang Y Wang W-B Wang A-P . PTH is a promising auxiliary index for the clinical diagnosis of aldosterone-producing adenoma. Am J Hypertens (2016) 29(5):575–81. doi: 10.1093/ajh/hpv146 26304960

[25] Jiang Y Zhang C Ye L Su T Zhou W Jiang L . Factors affecting parathyroid hormone levels in different types of primary aldosteronism. Clin Endocrinol (Oxf) (2016) 85(2):267–74. doi: 10.1111/cen.12981 26589237

[26] Petramala L Zinnamosca L Settevendemmie A Marinelli C Nardi M Concistrè A . Bone and mineral metabolism in patients with primary aldosteronism. Intl J Endocrinol (2014) 2014:6. doi: 10.1155/2014/836529 PMC4016829 24864141

[27] Ceccoli L Ronconi V Giovannini L Marcheggiani M Turchi M Boscaro G . Bone health and aldosterone excess. Osteoporos Int (2013) 24(11):2801–7. doi: 10.1007/s00198-013-2399-1 23695421

[28] Salcuni AS Palmieri S Carnevale V Morelli V Battista C Guarnieri V . Bone involvement in aldosteronism. J Bone Miner Res (2012) 27(10):2217–22. doi: 10.1002/jbmr.1660 22589146

[29] Rossi GP Ragazzo F Seccia TM Maniero C Barisa M Calò LA . Hyperparathyroidism can be useful in the identification of primary aldosteronism due to aldosterone-producing adenoma. Hypertension (2012) 60(2):431–6. doi: 10.1161/HYPERTENSIONAHA.112.195891 22733469

[30] Pilz S Kienreich K Drechsler C Ritz E Fahrleitner-Pammer A Gaksch M . Hyperparathyroidism in patients with primary aldosteronism: cross-sectional and interventional data from the GECOH study. J Clin Endocrinol Metab (2012) 97(1):E75–79. doi: 10.1210/jc.2011-2183 22013107

[31] Maniero C Fassinab A Secciaa TM Toniatod A Iacobonee M Plebanic M . Mild hyperparathyroidism: a novel surgically correctable feature of primary aldosteronism. J Hypertens (2012) 30(2):390–5. doi: 10.1097/HJH.0b013e32834f0451 22179087

[32] Rossi E Perazzoli F Negro A Sani C Davoli S Dotti C . Acute effects of intravenous sodium chloride load on calcium metabolism and on parathyroid function in patients with primary aldosteronism compared with subjects with essential hypertension. Am J Hypertens (1998) 11(1 Pt 1):8–13. doi: 10.1016/s0895-7061(97)00366-x 9504444

[33] Rossi E Sani C Perazzoli F Casoli MC Negro A Dotti C . Alterations of calcium metabolism and of parathyroid function in primary aldosteronism, and their reversal by spironolactone or by surgical removal of aldosterone-producing adenomas. Am J Hypertens (1995) 8(9):884–93. doi: 10.1016/0895-7061(95)00182-O 8541003

[34] Resnick LM Laragh JH . Calcium metabolism and parathyroid function in primary aldosteronism. Am J Med (1985) 78(3):385–90. doi: 10.1016/0002-9343(85)90328-6 3883768

[35] Atashi F Modarressi A Pepper MS . The role of reactive oxygen species in mesenchymal stem cell adipogenic and osteogenic differentiation: a review. Stem Cells Dev (2015) 24(10):1150–63. doi: 10.1089/scd.2014.0484 PMC4424969 25603196

[36] Cauley JA Danielson ME Boudreau RM Forrest KYZ Zmuda JM Pahor M . Inflammatory markers and incident fracture risk in older men and women: the health aging and body composition study. J Bone Miner Res (2007) 22(7):1088–95. doi: 10.1359/jbmr.070409 17419681

[37] Stehr CB Mellado R Ocaranza MP Carvajal CA Mosso L Becerra E . Increased levels of oxidative stress, subclinical inflammation, and myocardial fibrosis markers in primary aldosteronism patients. J Hypertens (2010) 28(10):2120–6. doi: 10.1097/HJH.0b013e32833d0177 20683341

[38] Carbone LD Cross JD Raza SH Bush AJ Sepanski RJ Dhawan S . Fracture risk in men with congestive heart failure risk reduction with spironolactone. J Am Coll Cardiol (2008) 52(2):135–8. doi: 10.1016/j.jacc.2008.03.039 18598893

[39] Beavan S Horner A Bord S Ireland D Compston J . Colocalization of glucocorticoid and mineralocorticoid receptors in human bone. J Bone Miner Res (2001) 16(8):1496–504. doi: 10.1359/jbmr.2001.16.8.1496 11499872

[40] Gupta M Cheung CL Hsu YH Demissie S Cupples LA Douglas PK . Identification of homogeneous genetic architecture of multiple genetically correlated traits by block clustering of genome-wide associations. J Bone Miner Res (2011) 26(6):1261–71. doi: 10.1002/jbmr.333 PMC3312758 21611967

[41] Brown J Williams JS Luther JM Garg R Garza AE Pojoga LH . Human interventions to characterize novel relationships between the renin-angiotensin-aldosterone system and parathyroid hormone. Hypertension (dallas tex. : 1979) (2014) 63(2):273–80. doi: 10.1161/HYPERTENSIONAHA.113.01910 PMC3898197 24191286

[42] Ismail NA Kamaruddin NA Azhar Shah S Sukor N . The effect of vitamin d treatment on clinical and biochemical outcomes of primary aldosteronism. Clin Endocrinol (Oxf) (2020) 92(6):509–17. doi: 10.1111/cen.14177 32073675

[43] Forman JP Williams JS Fisher NDL . Plasma 25-hydroxyvitamin d and regulation of the renin-angiotensin system in humans. Hypertension (2010) 55(5):1283–8. doi: 10.1161/HYPERTENSIONAHA.109.148619 PMC3023301 20351344

[44] Tomaschitz A Pilz S Ritz E Grammer T Drechsler C Boehm BO . Independent association between 1,25-dihydroxyvitamin d, 25-hydroxyvitamin d and the renin-angiotensin system: The ludwigshafen risk and cardiovascular health (LURIC) study. Clin Chim Acta (2010) 411(17–18):1354–60. doi: 10.1016/j.cca.2010.05.037 20515678

[45] Li YC Kong J Wei M Chen Z-F Liu SQ Cao L-P . 1,25-dihydroxyvitamin D(3) is a negative endocrine regulator of the renin-angiotensin system. J Clin Invest (2002) 110(2):229–38. doi: 10.1172/JCI15219 PMC151055 12122115

[46] Vaidya A Forman JP Hopkins PN Seely EW Williams JS . 25-hydroxyvitamin d is associated with plasma renin activity and the pressor response to dietary sodium intake in caucasians. J Renin Angiotensin Aldosterone Syst (2011) 12(3):311–9. doi: 10.1177/1470320310391922 PMC3146958 21330422

[47] Vaidya A Sun B Larson C Forman JP Williams JS . Vitamin D3 therapy corrects the tissue sensitivity to angiotensin ii akin to the action of a converting enzyme inhibitor in obese hypertensives: an interventional study. J Clin Endocrinol Metab (2012) 97(7):2456–65. doi: 10.1210/jc.2012-1156 PMC3387405 22539586

[48] Matrozova J Steichen O Amar L Zacharieva S Jeunemaitre X Plouin P-F . Fasting plasma glucose and serum lipids in patients with primary aldosteronism: a controlled cross-sectional study. Hypertension (2009) 53(4):605–10. doi: 10.1161/HYPERTENSIONAHA.108.122002 19221213

[49] Grubler MR Gaksch M Kienreich K Verheyen N Schmid J Hartaigh BWJO . Effects of vitamin d supplementation on plasma aldosterone and renin-a randomized placebo-controlled trial. J Clin Hypertens (Greenwich) (2016) 18(7):608–13. doi: 10.1111/jch.12825 PMC8032175 27098193

[50] Yuan W Pan W Kong J Zheng W Szeto FL Wong KE . 1,25-dihydroxyvitamin D3 suppresses renin gene transcription by blocking the activity of the cyclic AMP response element in the renin gene promoter. J Biol Chem (2007) 282(41):29821–30. doi: 10.1074/jbc.M705495200 17690094

[51] Kurpas A Supeł K Idzikowska K Zielińska M . FGF23: A review of its role in mineral metabolism and renal and cardiovascular disease. Dis Markers (2021) 2021:8821292. doi: 10.1155/2021/8821292 34055103 PMC8149241

[52] Ragazzo F Maniero C Seccia TM De Toni R Rossi GP . The phosphatonin FGF23 is associated with the subtle hyperparathyroidism of patients with primary aldosteronism due to an aldosterone-producing adenoma. High Blood Press Cardiovasc Prev (2012) 19(3):168. doi: 10.2165/11632200-000000000-00000

[53] Wu X Yu J Tian H . Cardiovascular risk in primary aldosteronism: A systematic review and meta-analysis. Med (Baltimore) (2019) 98(26):e15985. doi: 10.1097/MD.0000000000015985 PMC6617487 31261504

[54] Harvey NC Glüer CC Binkley N McCloskey EV Brandi M-L Cooper C. . Trabecular bone score (TBS) as a new complementary approach for osteoporosis evaluation in clinical practice. Bone (2015) 78:216–24. doi: 10.1016/j.bone.2015.05.016 PMC4538791 25988660

[55] Riester A Fischer E Degenhart C Reiser MF Bidlingmaier M Beuschlein F . Age below 40 or a recently proposed clinical prediction score cannot bypass adrenal venous sampling in primary aldosteronism. J Clin Endocrinol Metab (2014) 99(6):E1035–1039. doi: 10.1210/jc.2013-3789 24601689

[56] Rossi GP Ragazzo F Seccia TM Maniero C Barisa M Calò LA . Hyperparathyroidism can be useful in the identification of primary aldosteronism due to aldosterone-producing adenoma. High Blood Press Cardiovasc Prev (2012) 19(3):167. doi: 10.2165/11632200-000000000-00000 22733469

