# Supplementary figures and images for: Bone and mineral metabolism in patients with primary aldosteronism: A systematic review and meta-analysis
Source: Front Endocrinol (Lausanne). 2022 Oct 31;13:1027841. doi: 10.3389/fendo.2022.1027841 (PMC9659816; doi:10.3389/fendo.2022.1027841)

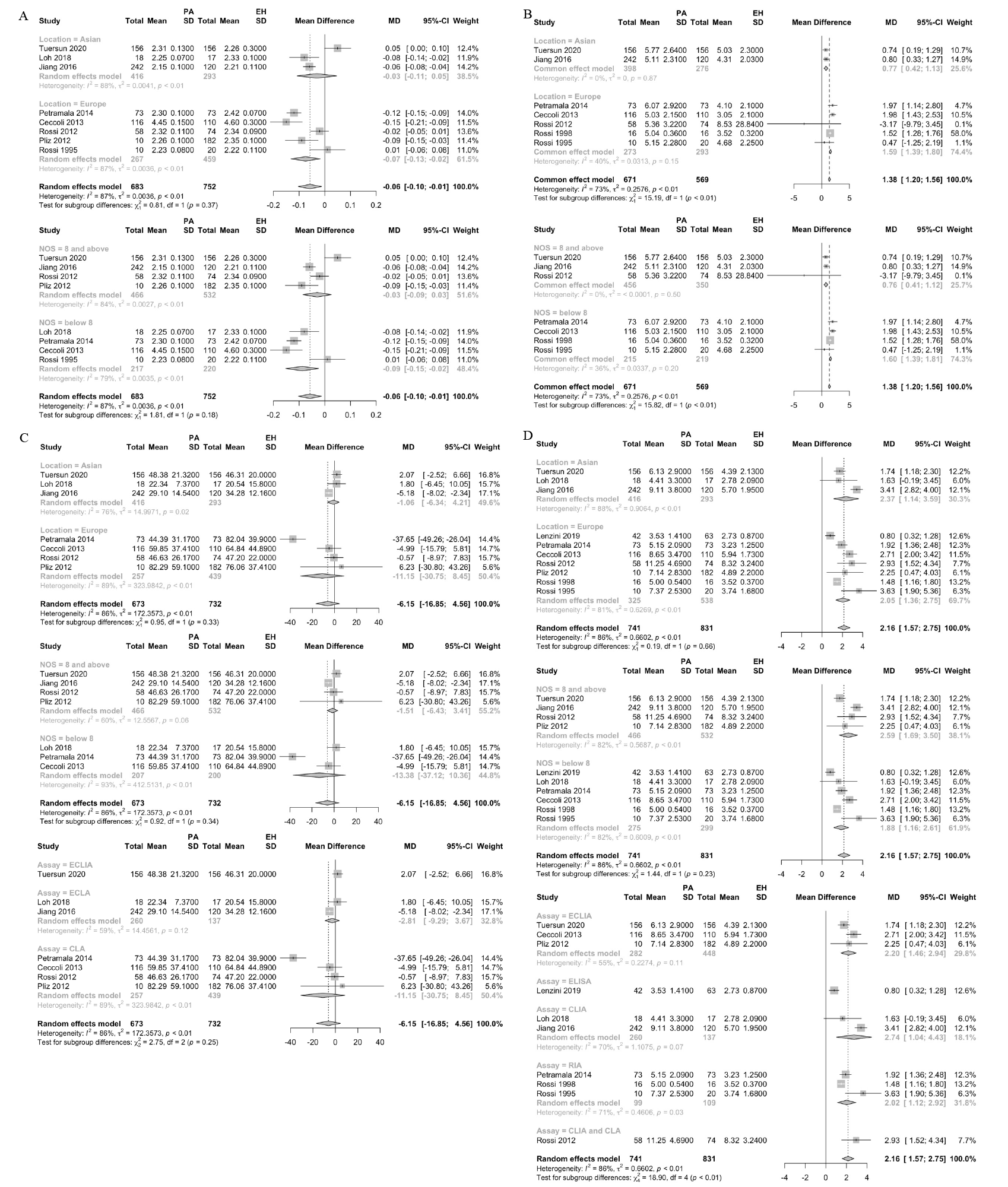

Supplement: Supplementary Figure 1 — Subgroup analysis of PA and EH (A: serum calcium, B: urine calcium, C: serum PTH, D: serum 25-OHD). [file Image_1.tif]

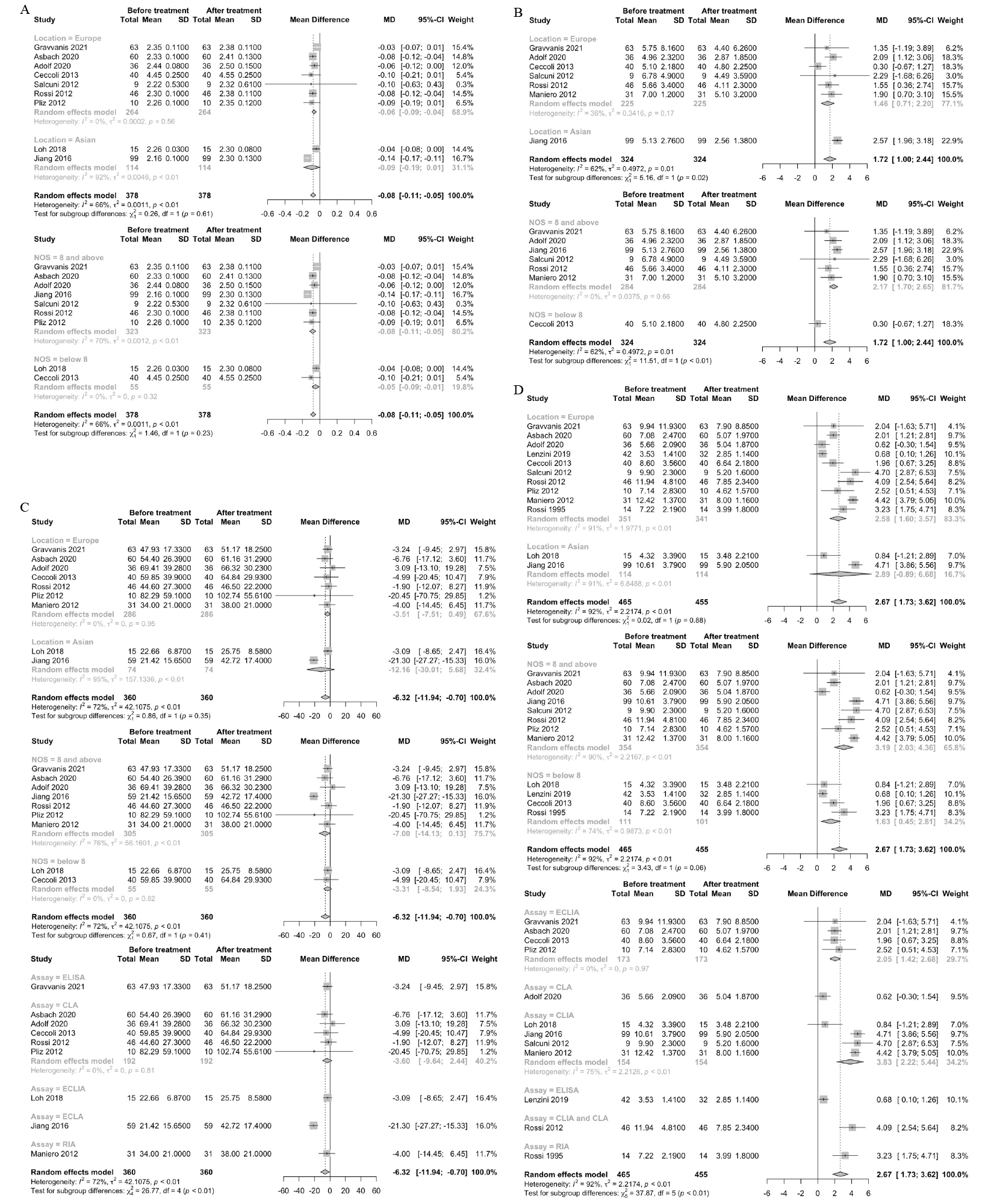

Supplement: Supplementary Figure 2 — Subgroup analysis of before-treatment and after-treatment PA patients (A: serum calcium, B: urine calcium, C: serum PTH, D: serum 25-OHD). [file Image_2.tif]

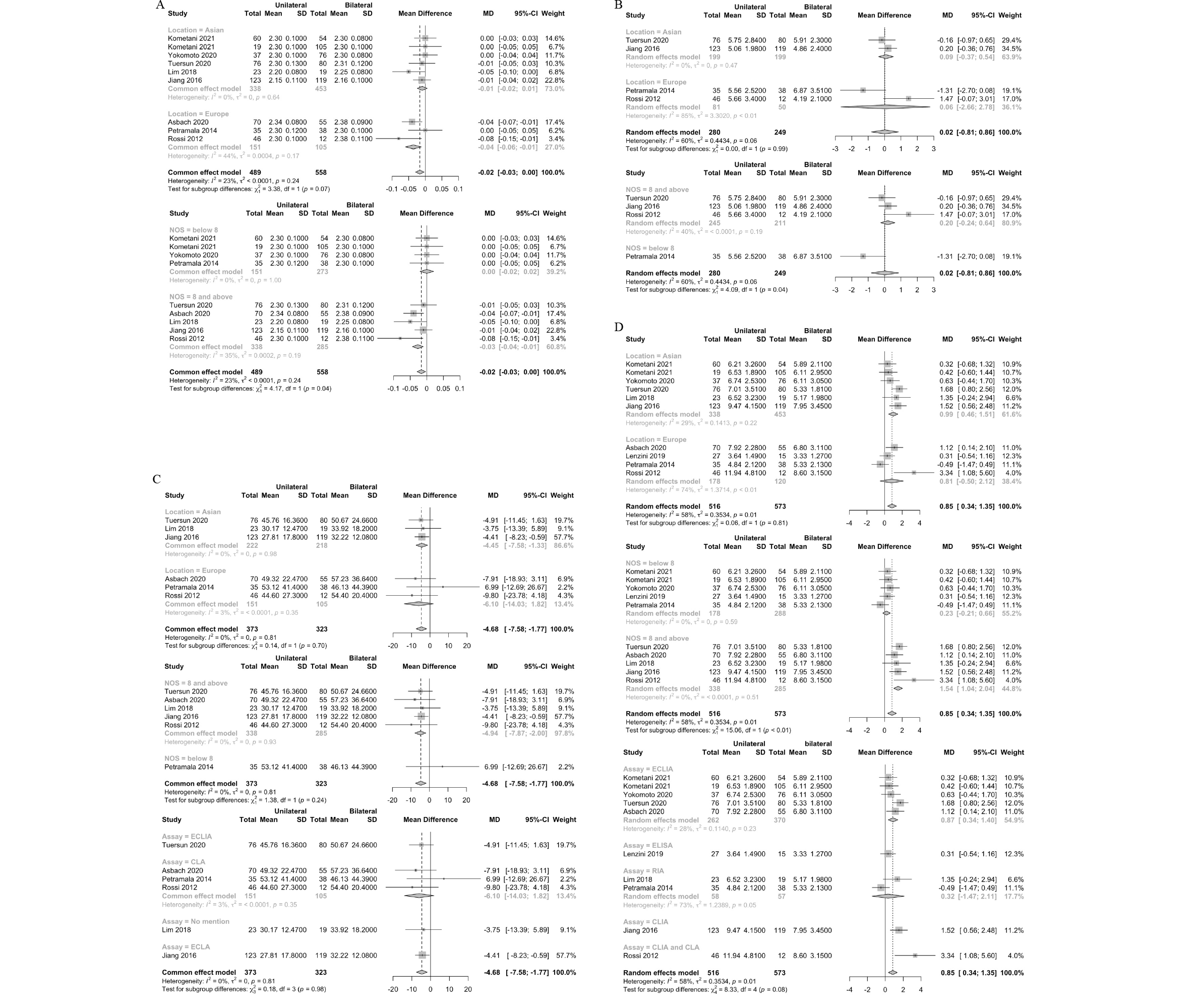

Supplement: Supplementary Figure 3 — Subgroup analysis of unilateral and bilateral PA patients (A: serum calcium, B: urine calcium, C: serum PTH, D: serum 25-OHD). [file Image_3.tif]
