# Supplementary material for: Bone and mineral metabolism in patients with primary aldosteronism: A systematic review and meta-analysis
Source: Front Endocrinol (Lausanne). 2022 Oct 31;13:1027841. doi: 10.3389/fendo.2022.1027841 (PMC9659816; doi:10.3389/fendo.2022.1027841)
Supplement: Supplementary file 4 [file DataSheet_1.docx]

Supplementary Material

# Supplementary Figures and Tables

## Supplementary Tables

**Supplementary Table 1.** PubMed search results.

| Search number | Query | Filters | Results |
| --- | --- | --- | --- |
| #1 | "Hyperaldosteronism"[Mesh] | - | 9335 |
| #2 | "osteoporosis"[Title/Abstract] OR "BMD"[Title/Abstract] OR "calcium"[Title/Abstract] OR "PTH"[Title/Abstract] | - | 497,653 |
| #3 | "Hyperparathyroidism, Secondary"[Mesh] | - | 8790 |
| #4 | #2 OR #3 | - | 502,306 |
| #5 | #1 AND #4 | - | 339 |
| #6 | #1 AND #4 | English, Humans | 270 |

**Supplementary Table 2**. Other clinical characteristics of studies included in systematic review and meta-analysis.

| Study | Patient and Control | BMI | SBP (mmHg) | DBP (mmHg) | PAC (ng/dL) | PRC (μIU/ml) | PRA (μg/l/h) | Phosphate (mmol/L) | PTH assay method | 25-OHD assay method |
| --- | --- | --- | --- | --- | --- | --- | --- | --- | --- | --- |
| Zavatta, 2022 | PA NFA | 27.7±5.0 28.1±4.5 | 148±23 139±17 | 89±10 85±8 | 29.80±20.70 21.80±13.80 | -  - | 0.28±0.11 1.25±1.32 | 1.00±0.19 1.10±0.19 | CLA | CLA |
| Tang, 2022 | PA SA(GS+BS) | 24.47±4.19 21.79±4.55 | -  - | -  - | 27.00 (16.90, 38.35) 16.90(12.25, 21.65) | 1.50(0.60, 2.40) 5.40(2.71, 34.55) | -  - | 1.09±0.19 1.24±0.21 | ECLA | ECLA |
| Liu, 2021 | PA NFA | 26±4 27±3 | -  - | -  - | -  - | -  - | -  - | 1.11±0.56 1.21±0.18 | ECLIA | ECLIA |
| Kometani, 2021 | (a) unilateral PA  bilateral PA | -  - | 134±17 137±18 | 85±12 85±14 | 22.30±23.30 16.70±8.00 | -  - | 0.50±0.50 0.60±0.20 | 1.10±0.19 1.13±0.16 | ECLIA | - |
|  | (b) unilateral PA  bilateral PA | -  - | 133±18 136±17 | 86±16 84±12 | 34.90±19.00 16.90±7.80 | -  - | 0.40±0.40 0.60±0.50 | 1.03±0.16 1.13±0.16 |  |  |
| Gravvanis, 2021 | PA before treatment PA after treatment | 31.44±7.22 | 150(125,230) 130(105,156) | 95(75,146) 80(65,95) | 16.90(2.94,94.29) 32.44(14.57,205.96) | 5.40(1.40,19.80) 11.70(1.80,65.88) | -  - | 0.99±0.16 1.04±0.18 | ECLIA | ELISA |
| Yokomoto, 2020 | unilateral PA bilateral PA | 24.6±3.8 25.0±3.8 | 142±16 139±19 | 87±12 87±13 | 32.70±18.80 18.50±9.00 | -  - | 0.20±0.10 0.40±0.30 | -  - | ECLIA | - |
| Tuersun, 2020 | (a) PA  EH | 26.57±4.19 26.75±4.09 | 148.17±17.23 148.60±22.72 | -  - | 19.76±6.68 16.08±4.76 | -  - | 0.55±0.38 2.24±1.90 | 1.09±0.17 1.09±0.21 | ECLIA | ECLIA |
|  | (b) unilateral PA  bilateral PA | 25.41±3.26 27.64±4.68 | 149.62±19.73 147.24±16.56 | 91.72±15.03 88.65±13.69 | 22.45±7.10 17.24±5.09 | -  - | 0.57±0.38 0.53±0.39 | 1.11±0.17 1.08±0.18 |  |  |
| Asbach, 2020 | (a) unilateral PA  bilateral PA | 28.0(25.8,30.9) 27.7(25.2,31.0) | 153(140,162) 152(135,169) | 94(87,103) 96(87,110) | 19.55(14.18,36.40) 16.00(10.70,22.10) | 3.90(2.00,6.20) 3.90(2.00,7.50) | -  - | -  - | ECLIA | CLA |
|  | (b) PA before treatment  PA after treatment | 28.64±4.01 28.35±3.92 | 149.74±20.44 133.08±18.56 | 94.98±15.89 86.38±9.71 | 20.97±15.98 15.18±16.29 | 3.92±3.51 17.05±18.77 | -  - | -  - |  |  |
| Adolf, 2020 | (a) PA  HS | 26.0(23.2,30.0) 27.0(25.9,34.8) | -  - | -  - | -  - | -  - | -  - | 1.07(0.94,1.19) 1.03(0.97,1.07) | CLA | CLA |
|  | (b) PA before treatment  PA after treatment | 26.0(23.2,30.0) 26.2(23.1,30.1) | -  - | -  - | -  - | -  - | -  - | 1.07(0.94,1.19) 1.13(0.97,1.19) |  |  |
| Lenzini, 2019 | (a) PA  EH | 25.69±4.25 25±3 | 149±19 143±14 | 92.64±12.51 91±6 | 30.06±57.87 8.40(1.50,15.00) | -  - | 1.16±3.58 0.50(0.10,4.40) | -  - | ELISA | - |
|  | (b) unilateral PA  bilateral PA | 25.4±4.1 26.2±4.6 | 149±18 150±22 | 93±13 92±12 | 14.20(4.70,93.70) 13.70(2.20,24.90) | -  - | 0.24(0.10,0.57) 0.24(0.10,7.16) | -  - |  |  |
|  | (c) PA before treatment  PA after treatment | 25.69±4.25 24.36±3.06 | 149.36±19.26 141.97±22.58 | 92.64±12.51 92.91±12.96 | 30.06±57.87 11.04±14.07 | -  - | 0.31±0.37 1.01±1.96 | -  - |  |  |
| Loh, 2018 | (a) PA  EH | 27.6(24.5,29.4) 27.8(25.9,32.1) | -  - | -  - | 24.26(14.98,40.38) 5.80(3.85,10.85) | -  - | 0.38(0.20,0.61) 1.30(0.71,2.15) | 1.18(1.03,1.22) 1.15(1.02,1.23) | CLIA | ECLIA |
|  | (b) PA before treatment  PA after treatment | -  - | -  - | -  - | -  - | -  - | -  - | 1.18(1.04,1.21) 1.18(1.08,1.32) |  |  |
| Lim, 2018 | unilateral PA bilateral PA | -  - | 149.7±18.8 148.9±18.6 | 89.8±13.1 92.0±11.1 | 42.60±27.00 23.20±12.10 | -  - | 0.23±0.22 0.25±0.26 | -  - | RIA | Unknown |
| Kim, 2018 | PA AI | 25.28±3.33 25.48±4.56 | 135.49±15.72 125.41±13.98 | 81.83±10.93 77.56±9.73 | 24.60±7.70 15.64±6.07 | -  - | 0.30±0.19 1.23±1.42 | -  - | - | - |
| Shu, 2018 | OP OE HS | 23.0±2.5 23.3±2.8 24.3±3.2 | 118.6±11.6 117.9±9.4 120.8±10.4 | 72.0±8.4 72.9±7.1 73.0±8.3 | 12.10±7.88 9.17±5.62 8.18±7.15 | 12.90±13.60 13.10±9.80 13.20±9.20 | -  -  - | -  -  - | CLIA | CLIA |
| Wu, 2017 | PA EH | -  - | -  - | -  - | -  - | -  - | -  - | -  - | - | - |
| Salcuni, 2017 | (a) PA  non-PA | 23.9(21) 25.6(30.6) | 140(90) 120(95) | 83(35) 75(55) | 8.76(15.14) 4.54(19.43) | -  - | -  - | 1.01±0.10 1.11±0.15 | CLIA | RIA |
|  | (b) OP  HS | -  - | -  - | -  - | -  - | -  - | -  - | -  - |  |  |
| Notsu, 2017 | PA HS | 24.70±4.00 23.50±6.30 | 144±21 131±18 | 85±14 75±13 | 19.94±13.96 - | -  - | 0.31±0.22 - | 1.10±0.19 1.07±0.16 | CLA | - |
| Zhang, 2016 | PA NFA | 26.00±3.30 26.70±7.70 | 149±20 133±20 | 93±12 83±14 | 20.36±7.38 11.08±3.22 | -  - | 0.27±0.62 1.11±1.55 | 1.04±0.18 1.18±0.19 | ECLIA | CLA |
| Jiang, 2016 | (a) PA  EH | 24.00(22.20,26.40) 25.50(23.0,28.00) | 180(160,190) 160(150,180) | 104(100,120) 100(90,110) | 48.55(34.06,68.23) 21.05(16.15,28.76) | -  - | 0.26(0.13,0.52) 0.78(0.39,2.60) | 1.10(0.95,1.20) 1.26(1.10,1.34) | CLIA | ECLA |
|  | (b) unilateral PA  bilateral PA | 23.14±2.65 25.52±3.15 | 177.24±27.21 172.97±15.01 | 105.67±7.68 103.87±21.76 | 61.28±36.45 39.50±17.20 | -  - | 0.31±0.29 0.40±0.49 | 1.07±0.17 1.08±0.19 |  |  |
|  | (c) PA before treatment  PA after treatment | -  - | 164.09±27.50 123.77±11.07 | 96.05±7.80 78.86±5.80 | 44.79±29.35 21.80±20.15 | -  - | 0.24±0.38 1.12±1.61 | 1.10±0.19 1.17±0.26 |  |  |
| Petramala, 2014 | (a) PA  EH  HS | 28.20±4.70 2900±5.00 25.10±2.20 | 138.3±16.8 131±18.8 119.1±4.2 | 85.9±11.4 82.4±11.2 77.2±5.1 | 37.00±25.10 22.50±13.00 9.20±1.70 | -  -  - | 0.90±0.70 1.40±1.60 1.10±0.40 | 1.13±0.19 1.10±0.13 1.10±0.10 | RIA | CLA |
|  | (b) unilateral PA  bilateral PA | 27.60±4.80 28.60±4.60 | 138.8±19.1 137.3±14.5 | 88.3±9.6 83.4±9.6 | 39.80±25.60 34.40±24.60 | -  - | 0.70±0.60 1.10±0.80 | 1.10±0.23 1.16±0.19 |  |  |
| Ceccoli, 2013 | (a) PA  EH | 27.80±4.80 30.10±5.40 | 158±19 151±15 | 97±11.2 93±7.5 | 4.98±3.40 1.60±1.08 | -  - | 0.40(0.20,0.70) 1.60±1.40 | -  - | ECLIA | CLA |
|  | (b) PA before treatment  PA after treatment | 27.70±4.90 28.0±4.20 | 153±17 138±21 | 97±10 84±11 | -  - | -  - | -  - | -  - |  |  |
| Salcuni, 2012 | (a) PA  NFA | 28.20±3.50 27.30±4.30 | 142±20 131±12 | 86±11 84±7 | 37.16(18.71,279.27) 24.69(16.11,55.29) | -  - | 0.21(0.01,0.53) 0.38(0.01,1.80) | 1.09±0.13 1.08±0.10 | CLIA | RIA |
|  | (b) PA before treatment  PA after treatment | -  - | -  - | -  - | -  - | -  - | -  - | -  - |  |  |
| Rossi, 2012 | (a) PA  EH | -  - | 155±20.94 149±16 | 93.59±12.22 93±15 | 18.60±10.29 13.50(9.40,10.40) | -  - | 0.73±0.76 1.92(0.98,6.20) | 1.00±0.15 0.91±0.13 | CLIA and CLA | CLA |
|  | (b) unilateral PA  bilateral PA | -  - | 155±17 155±33 | 94±10 92±19 | 19.00(12.20,26.70) 17.40(11.80,18.80) | -  - | 0.78(0.31,1.31) 0.20(0.15,1.00) | 0.99±0.14 1.06±0.20 |  |  |
|  | (c) PA before treatment  PA after treatment | -  - | 155±17 131±5 | 94±10 82±6 | 19.00(12.20,26.70) 9.00(6.70,13.50) | -  - | 0.78(0.31,1.31) 1.30(0.72,1.50) | 0.99±0.14 0.96±0.15 |  |  |
| Pilz, 2012 | (a) PA  EH | 31.00±7.10 28.50±6.00 | 179±22 154±23 | 108±12 94±13 | 33.60(24.40,67.80) 16.00(12.30,23.40) | 3.10(2.80,4.40) 11.90(5.90,28.20) | -  - | 0.81±0.22 0.94±0.18 | ECLIA | CLA |
|  | (b) PA before treatment  PA after treatment | 31.00±7.10 - | 179±22 149±23 | 108±12 96±13 | 33.60(24.40,67.80) 31.00(9.10,53.40) | 3.10(2.80,4.40) 16.10(10.20,24.10) | -  - | 0.81±0.22 1.00±0.16 |  |  |
| Maniero, 2012 | (a) PA  EH | -  - | 156±19 149±18 | 94±11 93±15 | 18.50(16.80,28.20) 11.10(9.60,14.90) | -  - | 0.57(0.21,1.00) 2.06(0.80,2.42) | -  - | CLIA | RIA |
|  | (b) PA before treatment  PA after treatment | -  - | -  - | -  - | 16.97(11.46,26.56) 9.05(7.13,13.28) | -  - | 0.57(0.22,0.80) 1.10(0.75,1.45) | 1.02±0.18 0.93±0.15 |  |  |
| Rossi, 1998 | PA EH | -  - | 161±3 157±3 | 105±1 102±1 | 29.10±3.10 18.40±4.40 | -  - | 0.20±0.04 1.14±0.35 | -  - | RIA | - |
| Rossi, 1995 | (a) PA  EH  HS | 23.10±0.80 24.10±1.26 24.30±1.50 | 168.4±15.3 163.75±13.52 121.0±12.2 | 102.70±2.50 102.45±4.22 72.30±5.30 | 37.20±11.40 20.85±8.97 19.30±10.00 | -  -  - | 0.10±0.02 0.87±0.66 1.28±0.96 | 2.99±0.44 2.69±0.56 2.77±0.36 | RIA | - |
|  | (b) PA before treatment  PA after treatment | -  - | 171.43±15.23 136.71±11.34 | 103.36±2.53 84.14±7.75 | 37.89±12.54 33.19±20.02 | -  - | 0.10±0.02 1.18±1.36 | -  - |  |  |
| Lawrence, 1985 | PA | - | - | - | - | - | - | - | - | - |

BMI: body mass index, SBP: systolic blood pressure, DBP: diastolic blood pressure, PAC: plasma aldosterone concentration, PRC: plasma renin concentration, PRA: plasma renin activity, NOS: Newcastle-Ottawa Scale, NFA: non-functioning adrenal tumour, SA: secondary aldosteronism, GS: gitelman syndrome, BS: bartter syndrome, AI: adrenal incidentaloma, OP: osteoporosis, OE: osteopenia, HS: healthy subjects, CLA: chemiluminescence assay, ECLA: electro-chemiluminescence assay, ECLIA: electro-chemiluminescence immunoassay, ELISA: enzyme linked immunosorbent assay, CLIA: chemiluminescent immunoassay, RIA: immunoradiometric assays.

**Supplementary Table 3.** Quality of case-control studies included in the systematic review and meta-analysis (NOS scale).

| Study | Adequate case definition | Representativeness of the cases | Selection of Controls | Definition of Controls | Study controls for age | Study controls for additional factors | Ascertainment of exposure | Same method of ascertainment for cases and controls | Non-Response rate | Total quality scores |
| --- | --- | --- | --- | --- | --- | --- | --- | --- | --- | --- |
| Zavatta, 2022 | 1 | 1 | 0 | 1 | 1 | 1 | 1 | 1 | 0 | 8 |
| Tang, 2022 | 1 | 1 | 0 | 1 | 1 | 0 | 1 | 1 | 0 | 7 |
| Liu, 2021 | 1 | 1 | 0 | 0 | 1 | 0 | 1 | 1 | 0 | 6 |
| Kometani, 2021 | 1 | 0 | 0 | 0 | 1 | 1 | 1 | 1 | 0 | 6 |
| Gravvanis, 2021 | 1 | 1 | 0 | 1 | 1 | 1 | 1 | 1 | 1 | 8 |
| Yokomoto, 2020 | 1 | 1 | 0 | 0 | 1 | 1 | 2 | 1 | 0 | 7 |
| Tuersun, 2020 | 1 | 1 | 1 | 1 | 1 | 1 | 1 | 1 | 0 | 8 |
| Asbach, 2020 | 1 | 1 | 0 | 1 | 1 | 1 | 1 | 1 | 1 | 8 |
| Adolf, 2020 | 1 | 1 | 1 | 1 | 1 | 1 | 1 | 1 | 0 | 8 |
| Lenzini, 2019 | 1 | 0 | 0 | 1 | 1 | 1 | 1 | 1 | 1 | 7 |
| Loh, 2018 | 1 | 1 | 0 | 1 | 1 | 0 | 1 | 1 | 1 | 7 |
| Lim, 2018 | 1 | 1 | 1 | 1 | 1 | 1 | 1 | 1 | 0 | 8 |
| Kim, 2018 | 1 | 1 | 1 | 1 | 1 | 0 | 1 | 1 | 1 | 8 |
| Shu, 2018 | 1 | 1 | 1 | 1 | 1 | 0 | 1 | 1 | 0 | 8 |
| Salcuni, 2017 | 1 | 1 | 1 | 1 | 1 | 1 | 2 | 1 | 0 | 9 |
| Notsu, 2017 | 1 | 1 | 1 | 1 | 1 | 0 | 1 | 1 | 1 | 8 |
| Zhang, 2016 | 1 | 0 | 0 | 1 | 0 | 0 | 2 | 1 | 1 | 6 |
| Jiang, 2016 | 1 | 1 | 1 | 0 | 1 | 0 | 2 | 1 | 1 | 8 |
| Petramala, 2014 | 1 | 1 | 0 | 0 | 1 | 1 | 1 | 1 | 0 | 6 |
| Ceccoli, 2013 | 1 | 1 | 1 | 1 | 1 | 0 | 1 | 1 | 0 | 7 |
| Salcuni, 2012 | 1 | 1 | 1 | 1 | 1 | 1 | 2 | 1 | 1 | 10 |
| Rossi, 2012 | 1 | 1 | 1 | 1 | 1 | 1 | 2 | 1 | 0 | 9 |
| Pliz, 2012 | 1 | 1 | 1 | 1 | 1 | 0 | 2 | 1 | 1 | 9 |
| Maniero, 2012 | 1 | 1 | 1 | 1 | 1 | 1 | 2 | 1 | 0 | 9 |
| Rossi, 1998 | 1 | 0 | 0 | 1 | 1 | 1 | 2 | 1 | 0 | 7 |
| Rossi, 1995 | 1 | 0 | 0 | 0 | 1 | 1 | 2 | 1 | 0 | 6 |
